# Carbon Monoxide Poisoning Effectively Treated with High-flow Nasal Cannula

**DOI:** 10.5811/cpcem.2019.9.43618

**Published:** 2019-11-19

**Authors:** Patrick Lee, Steven D. Salhanick

**Affiliations:** * Southern New Hampshire Medical Center, Department of Emergency Medicine, Nashua, New Hampshire; † Massachusetts/Rhode Island Center for Poison Control and Information, Staff Toxicologist, Boston, Massachusetts

## Abstract

Carbon monoxide (CO) poisoning is typically treated by administration of oxygen via non-rebreather mask (NRB). High-flow nasal cannula (HFNC) is an alternative to NRB in a variety of disease states. We report a case of the novel use of HFNC in the treatment of acute CO poisoning. A 29-year-old man presented with a carboxyhemoglobin (COHb) level of 29.8%. He was treated with HFNC, and COHb levels declined to 5.4% in 230 minutes. Given several theoretical advantages of HFNC relative to NRB, HFNC is a potential option for use in the treatment of CO poisoning.

## INTRODUCTION

Carbon monoxide (CO) poisoning is a significant cause of morbidity and mortality causing approximately 500 deaths annually, and as many as 25,000 hospitalizations per year.^1^,^2^ The treatment of CO poisoning is primarily aimed at removing CO through competitive binding of hemoglobin by administration of supplemental oxygen. In the majority of cases this is done using a non-rebreather mask (NRB). Alternatively, hyperbaric oxygen may be used in cases of more severe toxicity based on very high levels of carboxyhemoglobin (COHb) or signs or symptoms of severe toxicity.

Delivery of supplemental oxygen via high-flow nasal cannula (HFNC) has been used with increasing frequency. HFNC allows for delivery of a higher flow rate of oxygen as well as positive pressure and administration of warmed, humidified oxygen. Proponents suggest that it can be advantageous over other forms of supplemental oxygen delivery as it is often better tolerated than a NRB, can provide some degree of positive pressure, is useful in washing out anatomic dead space, and allows for up to 60 liters per minute (LPM) of oxygen administration.^3^ High flow rate of oxygen reduces the dilution of inspired oxygen that occurs when the volume of inspired gas exceeds the flow rate of the NRB. As such, HFNC provides some theoretical advantages over NRB for the treatment of CO poisoning. To date we find two recent reports of HFNC used to treat CO poisoning; however, widespread use has not been reported.^4^,^5^ Our case is submitted to support recent earlier findings and therefore support the novel use of HFNC following CO poisoning.

## CASE REPORT

A 29-year-old man presented to the emergency department (ED) after being found in an idling car parked in an enclosed space. The patient had last been seen approximately four hours prior to being found in the car. He was awake and alert upon arrival. He had significant nausea but no vomiting. He denied loss of consciousness, confusion, seizure, chest pain, shortness of breath or headache, or any concomitant ingestion. He had no significant medical or surgical history other than depression, but reported being a pack per day smoker. The patient had been transported from home via ambulance and administered 100% oxygen via NRB at a flow rate of 15 LPM for approximately 15 minutes during transport.

On arrival, the patient’s blood pressure was 130/63 millimeters of mercury (mmHg), pulse rate was 73 beats per minute, respiratory rate was 18 breaths per minute, oxygen saturation (SaO_2_) was 99% on NRB, and temperature was 36.6 degrees Celsius. He was awake and had normal mentation, and was in no apparent distress. Cardiovascular exam showed normal heart sounds, normal rhythm, no murmurs, rubs or gallops. Respiratory exam revealed no respiratory distress, equal and clear breath sounds bilaterally. Neurologically, he was oriented to name, place, time, and purpose. He had normal speech, Glasgow Coma Scale of 15, normal memory, cranial nerves II–XII, and motor and sensory exams were completely normal. His gait was normal. He had no nystagmus. His psychiatric exam revealed that he was feeling suicidal. Otherwise, he had an unremarkable physical exam.

Initial arterial blood gas (ABG) on room air shortly after arrival revealed a pH 7.369 [7.35–7.45], partial pressure of carbon dioxide (pCO_2_) of 41.9 mmHg (35–45 mmHg), partial pressure of oxygen (pO_2_) of 93.3 mmHg (75–100 mmHg), and COHb level of 29.8% (0–2%). His electrocardiogram, serum electrolytes, liver function tests, creatinine kinase, and complete blood count were within normal range, excepting a potassium level of 3.1 milliequivalents per liter (mEq/L) (3.5–5.1 mEq/L). Salicylate, troponin I, ethanol, and acetaminophen levels were below the assay limits of detection.

Immediately after obtaining the ABG results the patient was started on 100% oxygen via HFNC at 30 LPM due to concerns that he would not tolerate the NRB and could vomit due to complaints of severe nausea. His nausea resolved within the first hour of HFNC treatment. After 112 minutes of therapy via HFNC, a repeat ABG revealed the following: pH 7.403, COHb 11.5%, pCO_2_ 42.1 mmHg, and pO_2_ of 462.4 mmHg. It was felt that the patient was responding to HFNC therapy as evidenced by the decline in COHb and the increase in pO_2_. HFNC therapy at the same settings was continued for a total of approximately 230 minutes when a repeat ABG was obtained revealing a COHb of 5.4%. At this point the patient was asymptomatic and therapy was discontinued. Of note the patient did not receive antiemetic medication. Following administration of supplemental oral potassium he was transferred to the psychiatric service.

## DISCUSSION

HFNC is a promising treatment option for hypoxic respiratory failure in the ED due to the ability to more comfortably deliver oxygen at a high rate as well as deliver positive pressure and wash out anatomic dead space. HFNC delivers warmed, humidified oxygen at flow rates of up to 60 LMP and with positive pressure of 2–5 mmHg.^6^–^9^ The ability to warm the oxygen to core temperature and humidify at high flow rates also helps remove airway secretions, decreases work of breathing, and avoids drying out the airway, reducing epithelial injury.^10^ Current indications for HFNC in children include use for premature infants with respiratory distress syndrome and infants with bronchiolitis.^11^ The indications for HFNC in adults include non-hypercapnic hypoxemic respiratory failure requiring relatively high concentrations of inspired oxygen. Other applicable settings for HFNC include post- extubation support, postoperative respiratory failure, intubation support, tracheostomy weaning, and support of hypoxemic patients during fiberoptic bronchoscopy.^12^

**CPC-EM Capsule**

What do we already know about this clinical entity?

Carbon monoxide (CO) poisoning is effectively treated with supplemental oxygen, typically via a non-rebreather mask (NRB). In some clinical situations, however, patients may have difficulty tolerating the NRB.

What makes this presentation of disease reportable?

There are no prior reports of the use of high flow nasal cannula (HFNC) in the literature outside those reported using a strict study protocol. Our report supports effectiveness in clinical practice.

What is the major learning point?

HFNC is a likely effective option for the administration of supplemental oxygen when treating CO poisoning.

How might this improve emergency medicine practice?

Our report supports the use of HFNC which has distinct advantages over use of a NRB when administering supplemental oxygen to patients with elevated CO levels.

CO poisoning is treated with high levels of supplemental oxygen in an effort to displace CO from hemoglobin, other heme-containing molecules, and other sites of CO binding.^13^,^14^ This is typically realized clinically by the decrease in time that it takes to eliminate CO from hemoglobin in the peripheral circulation. At baseline CO is eliminated with a half-life of approximately 300 minutes.^15^ This can be reduced by oxygen administration via NRB to between 37–120 minutes.^16^ Given the proposed mechanism of elimination, it stands to reason that HFNC could be of similar or greater efficacy as it allows for higher oxygen flow rates than NRB.

There are several theoretical advantages to the use of HFNC in treating CO poisoning. HFNC can produce a higher partial pressure of oxygen than NRB when applied to patients who are tachypneic, potentially resulting in increased rate of elimination of CO relative to NRB. This is due to the phenomenon of mixing of ambient air into inspired air when the volume of air inspired exceeds the flow rate of oxygen delivered by a mask, typically 15 LPM. Given that HFNC can provide flow rates exceeding 60 LPM, the extent to which ambient air is mixed with supplemental oxygen is low; consequently, pO_2_ may be higher. HFNC is better tolerated than NRB due to improved comfort and warming and humidification of inspired gas, which may allow for better compliance with non-invasive oxygen therapy, particularly when administering to patients who may be agitated. Lastly, should vomiting occur, patients may be less likely to aspirate using HFNC as opposed to the NRB.

Previously, Tomruk et al. reported the use of HFNC routinely in a single academic medical center with an increase in the decline of absolute COHb levels in one hour compared to historical controls that received standard treatment with NRB. Mean levels of COHb were 22% in the HFNC group and 18.6% in the NRB group prior to treatment.^5^ Ozturan et al. reported a mean half-life of 36.8 minutes calculated from 33 patients enrolled with elevated COHb levels (mean 22.5% COHb prior to treatment) treated with HFNC.^4^ Neither study included any clinical outcome as endpoints. Our patient showed a decrease in COHb levels comparable to those described for NRB, indicating that HFNC delivered oxygen in a manner of comparable efficacy. Further, we were able to achieve a high pO_2_ with HFNC. Consequently, we would anticipate increased elimination of CO based on the pO_2_ achieved using HFNC.

While we did not have adequate data to calculate a half-life, our estimate based on the rate of decline of COHb between the first and second ABG suggests a half-life of approximately 81 minutes, and an estimate between the first and third COHb level suggests a half life of 92 minutes.^17^ These are consistent with reported half-lives using NRB and are exceeded more than four fold by the half-life generally reported with ambient air.^16^,^18^ The estimated half-life for our patient, however, was substantially less than that reported in prior studies using HFNC, suggesting that previous reports of half-life may not mirror effects in a uncontrolled clinical setting. Further, kinetics of elimination may vary as the initial COHb level in our patient was approximately 25% higher than the mean levels reported in the previous studies. Lastly, our patient reported a marked improvement in clinical symptoms following HFNC. Neither of the prior reports noted any clinical outcome. This is significant as early and late clinical findings may not correlate with COHb levels.^19^

There are several limitations to the data. First, we did not measure clearance directly and thus cannot state unequivocally that our half-life estimates are correct. However, given the substantial difference between our estimate of half-life and the half-life expected on room air, as well as the directly measured increase in arterial pO_2_, we feel confident that HFNC had a therapeutic effect. Another issue may be that we measured an artificially high initial level of COHb as there is an initial distribution phase of CO. We think this is unlikely as the time lapse between removal from the exposure and obtaining blood gas was greater than 30 minutes. Given that the initial rate of distribution is rapid we think that our initial COHb level likely represents a steady state level.^19^,^20^ Also, while our patient’s nausea resolved, we have no information regarding any long-term clinical issues or whether HFNC administration may have any effect on these given the limitations of COHb levels in predicting clinical outcome. Further, we cannot say this approach would have better outcome that use in NRB and antiemetics. Lastly, our patient was a chronic tobacco smoker, affecting his baseline levels of COHb and possibly his tolerance for elevated COHb levels.

## CONCLUSION

In a patient with a CO poisoning, as evidenced by symptoms and COHb level, use of HFNC resulted in an apparent increase in the rate of elimination of COHb similar to that seen with administration of oxygen via NRB. HFNC was well tolerated and may be a good option for treatment of CO poisoning.
